# A 26-week, randomized trial of insulin detemir versus NPH insulin in children and adolescents with type 2 diabetes (iDEAt2)

**DOI:** 10.1007/s00431-018-3205-z

**Published:** 2018-07-16

**Authors:** Mark D. Wheeler, Margarita Barrientos-Perez, Fu-Sung Lo, Bo Liang, Alison Lunsford, Ólöf Thórisdóttir, Nehama Zuckerman-Levin

**Affiliations:** 1 The University of Arizona Department of Pediatrics, 1501 N. Campbell Avenue, Room 3301, PO Box 245073, Tucson, AZ 85724 USA; 2 Servicio de Endocrinología Pediátrica, Hospital Angeles de Puebla, Av. Kepler 2143, Cons. 715 B, Col. Reserva Territorial Atlixcayotl, Puebla, CP 72190 Puebla, Mexico; 3 Division of Pediatric Endocrinology & Genetics, Department of Pediatrics, Chang Gung Memorial Hospital, Chung Gung University College of Medicine, No. 5 Fusing Street, Gueishan, Taoyuan County, 333 Taiwan; 4 Novo Nordisk A/S, Vandtårnsvej 114, 2860 Søborg, Denmark; 5 Department of Pediatrics, Texas Tech University Health Sciences Center, 1400 S Coulter Street, Amarillo, TX 79106 USA; 6 Pediatric Diabetes Clinic, Institute of Endocrinology, Diabetes and Metabolism, Rambam Health Care Campus, 31096 Haifa, Israel

**Keywords:** Type 2 diabetes, Insulin detemir, Adolescents, NPH insulin, Children

## Abstract

There are limited studies evaluating the safety and efficacy of treatments in young people with type 2 diabetes (T2D). This study compared the efficacy and safety of insulin detemir versus neutral protamine Hagedorn (NPH) insulin, both in combination with metformin and lifestyle intervention, in children and adolescents with T2D. This randomized, open-label, phase 3 trial recruited patients (*n* = 42) aged 10–17 years diagnosed with T2D already receiving metformin ± other oral antidiabetic drugs ± basal insulin. Patients were randomized (1:1) to receive either insulin detemir or NPH insulin, both with the maximum tolerated dose of metformin, and lifestyle intervention, over 26 weeks. Enrollment terminated prematurely after 17 months due to a very slow recruitment rate (12% of the target met). After 26 weeks, the observed mean HbA_1c_ value had decreased by 0.61% points in the insulin detemir group vs. 0.84% points in the NPH insulin group. The rate of symptomatic blood glucose-confirmed hypoglycemic episodes was 0.4 episodes/patient-year of exposure (PYE) for insulin detemir vs. 1.1 episodes/PYE for NPH insulin.

*Conclusion:* No safety issues were revealed with either basal insulin. Due to the low number of patients recruited, no efficacy conclusions could be drawn.

*ClinicalTrials.gov* *identifier*: NCT02131272.

**Table:** 

**What is known:** *• There is a growing worldwide epidemic of type 2 diabetes in children and adolescents.* *• There is a lack of research and limited treatment options currently available in this population.*	
**What is new:** *• No safety issues with insulin detemir or neutral protamine Hagedorn insulin in children and adolescents with type 2 diabetes were observed.* *• Improving clinical trial recruitment, along with providing early, efficacious, and safe treatment options, in this population is critical.*

**Electronic supplementary material:**

The online version of this article (10.1007/s00431-018-3205-z) contains supplementary material, which is available to authorized users.

## Introduction

Type 2 diabetes (T2D) is considered a growing epidemic in children and adolescents [2], due to factors such as the high prevalence of obesity in young people [26]. According to worldwide data, the estimated incidence of T2D is currently 330 per 100,000 person-years, and the estimated prevalence is 5300 per 100,000 population [4]. In recent years, the USA has witnessed a rapid increase in T2D in youth [15], with approximately 5000 new cases every year [13], and a projected fourfold rise in the number by 2050 [9].

T2D is a progressive disease characterized by hyperglycemia, insulin resistance, and reduced insulin secretion by beta-cells in the pancreas [10]. The rate at which insulin secretion by beta-cells diminishes in T2D is greater in youth (age 10–17 years) than in adults [7], which results in earlier and more aggressive development of diabetes-related complications [3, 18].

First-line management of T2D in children and adolescents includes dietary and exercise lifestyle modifications. Metformin and/or basal insulin are recommended by the American Diabetes Association (ADA) 2018 guidelines and the International Society for Pediatric and Adolescent Diabetes when lifestyle modification alone is insufficient to improve glycemic control [1, 27]. The failure of metformin treatment to maintain glycemic control, and thus, the need to consider starting insulin treatment, occurs sooner in youth than adults due to the rapid progression of T2D in youth [24, 25]. Despite a variety of insulins being used to treat youth-onset T2D, there have been no studies to evaluate insulin for treating the distinct indication of pediatric T2D [16].

Insulin detemir has been shown to offer a weight-sparing effect, improve glycemic control, and reduce the number of hypoglycemic episodes compared with neutral protamine Hagedorn (NPH) insulin [8, 20] in adults with T2D. Furthermore, insulin detemir has previously been shown to be effective and safe in type 1 diabetes in youth as part of a basal–bolus regimen [22], and as such, it may prove beneficial in children and adolescents with T2D. The current trial was conducted to compare the benefits and risks of insulin detemir versus NPH insulin, in combination with metformin, in children and adolescents with T2D with inadequate glycemic control.

## Methods

### Design

This 26-week, open-label, randomized, two-armed, parallel-group, multicenter, phase 3 trial recruited patients aged 10–17 years with T2D who had insufficient glycemic control with the maximum tolerated dose (MTD) of metformin ± other oral antidiabetic drugs (OADs) ± basal insulin. The study ran between June 2014 and June 2016, screening individuals from Brazil, Hungary, Germany, India, Israel, South Korea, Malaysia, Mexico, Russia, Taiwan, Turkey, and the USA (ClinicalTrials.gov identifier: NCT02131272). Written consent was collected from all patients or legally acceptable representatives. An independent data-monitoring committee reviewed and evaluated accumulating safety data from the trial to protect the safety of the patients and evaluate the benefit-versus-risk assessment. The trial was conducted according to the Declaration of Helsinki, Good Clinical Practice (International Conference on Harmonisation), and US Food and Drug Administration Code of Federal Regulations Title 21 312.120.

The study included male and female patients with a diagnosis of T2D at least 3 months prior to screening, and HbA_1c_ ≥ 7.0% and ≤ 10.5% (≥ 53 and ≤ 91 mmol/mol) at the time of screening. Patients were treated with the MTD of metformin for at least 3 months prior to screening or had documented complete metformin intolerance. Other OADs and basal insulin were allowed, and bolus insulin was only allowed as rescue treatment for a maximum of 7 days for the last 3 months prior to screening.

Key exclusion criteria were the presence of known or suspected hypersensitivity to trial products, maturity-onset diabetes of the young, impaired liver function (alanine aminotransferase ≥ 2.5 times the upper limit), known proliferative retinopathy or maculopathy requiring acute treatment, and pregnancy, breastfeeding, or willingness to become pregnant. Furthermore, the trial excluded patients who had been treated with any medication other than metformin ± other OADs ± basal insulin for the indication of diabetes or obesity within 3 months prior to screening.

After an initial 2-week screening period, eligible patients were randomly assigned to either insulin detemir or NPH insulin (1:1 ratio), both in combination with metformin and recommended lifestyle interventions (i.e., diet and exercise) for 26 weeks. A diet and exercise intervention, which followed a family-based behavioral weight-loss approach, was introduced not only to improve glycemic control, but also to motivate patients throughout the study, and was inspired by the educational material used in the TODAY study [23, 25]. The total daily dose of metformin was not changed, unless for safety reasons. Treatment with other OADs was discontinued at randomization.

Insulin detemir 100 U/mL and NPH insulin 100 IU/mL were supplied in a 3 mL pre-filled FlexPen (Novo Nordisk, Bagsvaerd, Denmark) and administered subcutaneously once or twice daily. For insulin-naive patients, insulin detemir and NPH insulin were initiated at a dose of 0.1–0.2 U/kg, with a maximum dose of 10 U, at the investigators’ discretion. Patients who were already receiving basal insulin before the trial were switched to equivalent units of insulin detemir or NPH insulin and maintained their pre-trial daily injection frequency. Insulin detemir and NPH insulin doses were titrated to a target self-measured blood glucose (SMBG) of 4.0–6.0 mmol/L (71–108 mg/dL), based on average pre-breakfast or pre-dinner SMBG measurements taken on any 3 days in the week prior to a site visit/phone contact (Supplementary Table 1).

### Endpoints

The primary efficacy endpoint was change in HbA_1c_ from baseline after 26 weeks of treatment. The secondary efficacy endpoints, measured at week 26, were the number of patients achieving HbA_1c_ < 7.0% (53 mmol/mol) (total and without treatment-emergent severe hypoglycemic episodes within the last 14 weeks of treatment) and < 7.5% (58 mmol/mol), and the mean of the 7-point SMBG profile. Additional secondary efficacy endpoints included change from baseline to week 26 in fasting plasma glucose (FPG), body weight standard deviation score (SDS), height SDS, body mass index (BMI), and BMI SDS.

Safety endpoints included incidence of adverse events (AEs) and hypoglycemic events during 26 weeks of treatment. Both total and nocturnal (occurring between 2300 and 0659 hours) treatment-emergent severe (requiring assistance) or blood glucose (BG)-confirmed (BG <3.1 mmol/L [< 56 mg/dL]) symptomatic episodes were reported.

### Statistics

The sample size was determined to show non-inferiority of insulin detemir to NPH insulin, in combination with the MTD of metformin and diet/exercise intervention, in change in HbA_1c_ from baseline after 26 weeks of treatment, using a non-inferiority limit of 0.4%. A total of 358 patients were initially planned to be randomized in this trial to have 80% power and show non-inferiority for both the full analysis set (FAS; i.e., all randomized patients) and the per-protocol (PP) analysis set (i.e., all randomized patients treated for at least 12 weeks and not violating any of the inclusion/exclusion criteria). However, due solely to a very slow recruitment rate, the sponsor decided to stop enrollment at 17 months and offered the 42 recruited patients the opportunity to complete the trial.

Formal statistical analysis was conducted on change in HbA_1c_ from baseline to 8, 16, and 26 weeks using a mixed model for repeated measurements with treatment, age group (two levels: 10–14 years and 15–17 years), prior antidiabetic therapy (two levels: metformin only or metformin in combination with other OAD[s] and/or basal insulin), and the interaction between age group and prior antidiabetic therapy as factors, and baseline HbA_1c_ as a covariate, with all variables nested within week as a factor. The SDS were derived by comparing the measurements taken in the trial with standard growth charts for the USA [12].

As a result of the limited number of patients included in the trial, the planned statistical analysis on the secondary efficacy and safety endpoints was not conducted and therefore only descriptive statistics are used to report these results. The descriptive statistics reported at week 26 are based on last observation carried forward (LOCF).

## Results

A total of 71 patients were screened, of whom 29 were screening failures mostly due to not meeting the inclusion criterion of HbA_1c_ 7.0–10.5% (53–91 mmol/mol). Of the 42 patients who were randomized, 39 completed the trial (Supplementary Fig. 1). Baseline characteristics are reported in Table 1; differences noted between the two groups were not unexpected due to the small number of patients recruited.

**Table 1:** Baseline characteristics

Characteristic	Insulin detemir	NPH insulin
FAS, *n*	20	22
Sex, *n* (%)		
Male	8 (40)	7 (31.8)
Female	12 (60)	15 (68.2)
Age		
10–14 years	9	11
15–17 years	11	11
Diabetes duration, years	2.3 (1.9)	3.3 (1.7)
Body weight, kg	75.9 (16.6)	73.2 (23.4)
Body weight, SDS	1.5 (0.7)	1.3 (0.8)
BMI, kg/m^2^	28.7 (4.8)	27.7 (6.6)
BMI, SDS	1.7 (0.5)	1.5 (0.7)
Height, m	1.62 (0.08)	1.61 (0.10)
Height, SDS	−0.09 (1.02)	−0.17 (1.04)
Hispanic or Latino, *n* (%)	5 (25.0)	10 (45.5)
White/Black/Asian/American Indian or Alaska native/other, %	40/0/55/0/5	50/4.5/31.8/4.5/9.1
HbA_1c_, %	8.7 (0.9)	9.0 (1.1)
FPG, mmol/L	8.0 (2.5)	10.2 (3.5)
Antidiabetic treatment at screening, n (%)		
Metformin only	4 (20)	5 (23)
Metformin + basal ± OAD	16 (80)	17 (77)

Data are arithmetic means (SD), unless stated otherwise

*FAS* full analysis set, *FPG* fasting plasma glucose, *NPH* neutral protamine Hagedorn, *OAD* oral antidiabetic drug, *SD* standard deviation, *SDS* standard deviation score

### Efficacy

**Fig. 1:**
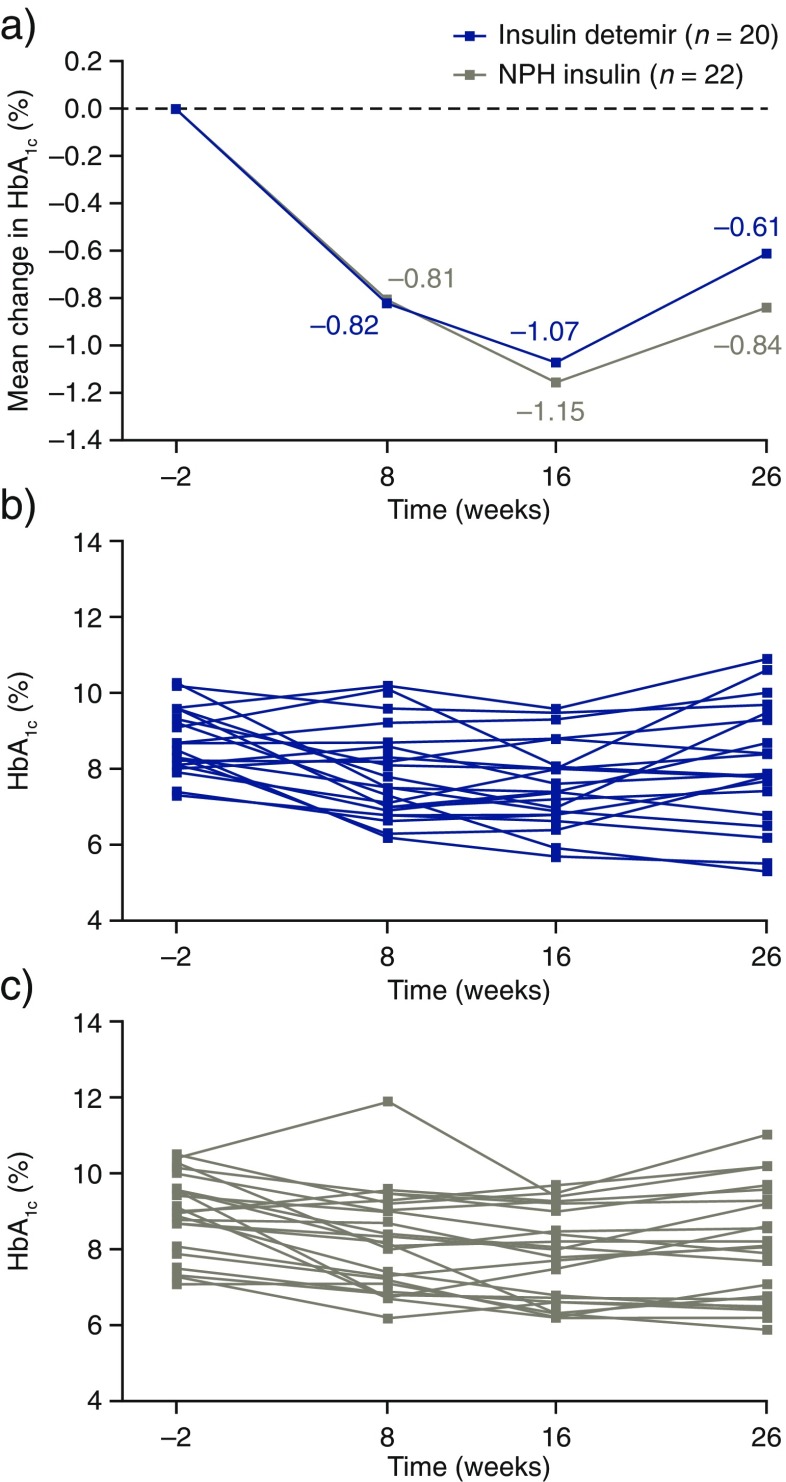
**a** Mean change in HbA_1c_ over time, and individual patient plots for HbA_1c_ over time in **b** insulin detemir and **c** NPH insulin groups by week of treatment. *NPH* neutral protamine Hagedorn

Observed mean HbA_1c_ decreased largely in the first 16 weeks in both treatment groups (Fig. 1). Median values and range (min; max) for HbA_1c_ with insulin detemir and NPH insulin by week of treatment are shown in Table 2. After 26 weeks of treatment, mean HbA_1c_ had decreased by 0.61% points (6.7 mmol/mol) to 8.11% (65 mmol/mol) in the insulin detemir group and by 0.84% points (9.2 mmol/mol) to 8.11% (65 mmol/mol) in the NPH insulin group. The estimated mean treatment difference at week 26 was 0.17% (95% confidence interval − 0.74; 1.09; *p* = 0.3075); however, no efficacy conclusions could be drawn from the primary analysis due to the low number of patients included in the trial.

**Table 2:** Summary of HbA_1c_ (%) by visit and treatment

Time from randomization	HbA_1c_, %	
	Insulin detemir	NPH insulin
Week –2	8.6 (7.3; 10.3)	9.0 (7.1; 10.5)
Week 8	7.7 (6.2; 10.2)	8.1 (6.2; 11.9)
Week 16	7.5 (5.7; 9.6)	7.8 (6.2; 9.7)
Week 26	7.9 (5.3; 10.9)	8.1 (5.9; 11.0)

Data are median (min; max)

*FAS* full analysis set, *NPH* neutral protamine Hagedorn

Secondary efficacy endpoints after 26 weeks of treatment are reported in Table 3. Observed mean FPG values decreased during the 26-week treatment period in both groups, with a numerically smaller change from baseline in the insulin detemir group compared with NPH insulin (− 0.335 vs. − 2.332 mmol/L, respectively), largely accounted for by the higher baseline FPG value in the NPH insulin group compared with the insulin detemir group. As expected in this trial population [14], observed mean body weight increased in both treatment groups; however, weight gain was numerically lower in the insulin detemir group (1.89 kg) compared with the NPH insulin group (4.00 kg).

**Table 3:** Secondary efficacy endpoints after 26 weeks of treatment

Endpoint	Insulin detemir (*n* = 20)	NPH insulin (*n* = 22)
FPG, mmol/L	7.66 (2.63)	7.86 (2.88)
Change from baseline	− 0.335 (2.961)	− 2.332 (4.147)
Body weight, SDS	1.538 (0.690)	1.358 (0.840)
Change from baseline	0.006 (0.192)	0.098 (0.139)
Body weight, kg	77.79 (16.16)	77.20 (24.60)
Change from baseline	1.89 (3.40)	4.00 (3.75)
Height, SDS	− 0.129 (1.057)	− 0.185 (1.046)
Change from baseline	− 0.042 (0.167)	− 0.019 (0.152)
BMI, kg/m^2^	29.09 (4.40)	28.90 (7.20)
Change from baseline	0.35 (1.22)	1.20 (1.29)
BMI, SDS	1.694 (0.405)	1.538 (0.658)
Change from baseline	0.013 (0.176)	0.086 (0.132)
Mean 7-point SMBG^a^, mmol/L	8.28 (2.72)	8.45 (3.06)
Patients achieving HbA_1c,_ *n* (%)		
< 7.0% (< 53 mmol/mol)	5 (25)	7 (31.8)
< 7.5% (< 58 mmol/mol)	6 (30)	8 (36.4)
Patients achieving HbA_1c_ < 7.0% (< 53 mmol/mol) without treatment-emergent severe hypoglycemic episodes^b^, *n* (%)	5 (25)	7 (33.3)

Data are mean (SD) unless specified

*BMI* body mass index, *FPG* fasting plasma glucose, *NPH* neutral protamine Hagedorn, *SDS* standard deviation score, *SMBG* self-measured blood glucose

^a^Mean 7-point SMBG profile is calculated as the arithmetic mean of all 7 points

^b^Within the last 14 weeks of treatment and only subjects who had been exposed for minimum 14 weeks included

The mean insulin dose at baseline was 25.2 U (0.342 U/kg) and 16.2 IU (0.226 IU/kg) in the insulin detemir and NPH insulin groups, respectively. After 26 weeks of treatment, the mean basal insulin dose had increased to 69.6 U (0.884 U/kg) in the insulin detemir group and 65.4 IU (0.818 IU/kg) in the NPH insulin group.

### Safety

No severe hypoglycemic episodes were reported and the rate of symptomatic BG-confirmed hypoglycemia was low in both treatment groups (Table 4).

**Table 4:** Treatment-emergent hypoglycemic events

	Insulin detemir (*n* = 20)			NPH insulin (*n* = 22)		
	*N* (%)	E	R	*N* (%)	E	R
Severe	0			0		
Symptomatic BG-confirmed	1 (5.0)	4	0.4	5 (22.7)	12	1.1
Nocturnal severe	0			0		
Nocturnal symptomatic BG-confirmed	0			1 (4.5)	1	0.1

Hypoglycemia was classified as “severe” (American Diabetes Association classification) if the episode required assistance of another person to actively administer carbohydrates, glucagon, or take other corrective actions, and as “symptomatic BG-confirmed” (Novo Nordisk classification) if the episode was not severe and with a plasma glucose value < 3.1 mmol/L (< 56 mg/dL) with symptoms. Nocturnal episodes are the episodes reported with the time of onset between 2300 and 0659 hours, both inclusive

*%* percentage of patients, *BG* blood glucose, *E* number of episodes, *N* number of patients, *NPH* neutral protamine Hagedorn, *R* event rate per patient-year of exposure

During 26 weeks of treatment, a total of 30 AEs were reported in eight patients in the insulin detemir group (rate 3.1 events/PYE) and 41 AEs were reported in 13 patients in the NPH insulin group (rate 3.9 events/PYE). The most frequently reported AEs (in 10–15% of participants in either of the two treatment groups) were gastroenteritis, headache, oropharyngeal pain, pyrexia, and vomiting. No AEs led to withdrawal from the trial. One serious AE (i.e., migraine) was reported in the NPH insulin group that was moderate in severity and was considered unlikely to be related to the trial product. No deaths were reported.

There were no clinically relevant changes in the development of anti-insulin antibodies in either of the treatment groups from baseline to week 26.

## Discussion

This trial was conducted to evaluate the efficacy and safety of insulin detemir in combination with metformin in children and adolescents with T2D with inadequate glycemic control. The initial plan was to enroll a total of 358 patients; however, due to a very slow recruitment rate indicating that completion of the trial within a relevant timeframe would not be possible, it was decided to stop further enrollment after 17 months. This resulted in the recruitment of 42 patients. No efficacy conclusions can be drawn given the low number of recruited patients, but the study did not present any new safety issues.

From baseline to 26 weeks after start of treatment, the mean change in both HbA_1c_ and FPG decreased for both the insulin detemir and NPH insulin groups. The observed trend in HbA_1c_ reduction seemed to decline after 16 weeks in both treatment groups. Although no efficacy conclusions can be drawn due chiefly to the low number of patients recruited, the upward trend in HbA_1c_ after 16 weeks may be attributable to treatment-independent factors, such as rapid beta-cell deterioration or trial fatigue. The difference in the change from baseline in FPG may have been driven by the different mean FPG values observed between the treatment groups at baseline (i.e., the higher baseline FPG value observed in the NPH insulin group). Additionally, considering the limitations of the study, the rate of severe or BG-confirmed hypoglycemic episodes was numerically lower in the insulin detemir versus the NPH insulin group. These efficacy and safety findings are aligned with what has been observed in studies in adults with T2D [5].

Due to the often rapid deterioration of glycemic control witnessed in T2D in youth compared with adults [16], providing early, efficacious, and safe treatment options is critical. This need is exacerbated when clinicians are reluctant to start insulin treatment in people with T2D (i.e., “clinical inertia”) [11]. Although the low number of patients included in this trial prevents the drawing of firm conclusions, the results may provide clinically relevant insights into the management of T2D in children and adolescents.

It is important to discuss the wider context of this clinical trial. Specifically, recruitment is problematic in clinical trials involving children and adolescents with T2D, mainly due to poor engagement [16, 17, 21]. For instance, previous analyses have indicated that clinical trial recruitment is generally low in this population and can range from 3.3 to 65% [17]. While the recruitment rate of the current trial was low (12% in 17 months), retention (93%) was excellent, ranking at the higher end of the range of results reported from other studies (74–100%) [6, 17]. This high retention may be explained by the relatively short duration of the study and the inclusion of a lifestyle program that involved the patient’s family and was based on a behavioral weight-loss approach. The TODAY study, which used a very similar interventional lifestyle program, showed a reduction in treatment adherence over 5 years across all treatment groups, as well as a reduction in attendance rate at scheduled lifestyle program visits from 75.2% in the first 2 years to 53.6% thereafter [25]. However, patients who had a lifestyle program as an add-on to metformin were at least 10% more adherent to their medication compared with those patients who were treated with metformin alone, or with metformin plus rosiglitazone, suggesting that a family-based behavioral program may help improve trial retention [25]. The higher retention observed in our study may have also been partly driven by the care and monitoring required when intensifying with basal insulin, which was not available as a treatment option in the TODAY trial.

To improve outcomes for this population, increasing the recruitment period of clinical trials may help researchers attract the required number of patients; however, there are wider issues that should also be addressed. Indeed, poor engagement in clinical trials is possibly indicative of the failure of this population to engage with routine clinical care [19], and it can be argued that this population requires a holistic/multidisciplinary approach to promote engagement and improve outcomes. To conclude, no new safety issues with insulin detemir or NPH insulin in children and adolescents with T2D were observed.

## Electronic supplementary material

ESM 1 (PDF 195 kb)

## Abbreviations

ADA: American Diabetes Association
AE: adverse event
BG: blood glucose
BMI: body mass index
E: number of episodes
FAS: full analysis set
FPG: fasting plasma glucose
IDet: insulin detemir
IU: international unit
LOCF: last observation carried forward
MTD: maximum tolerated dose
NPH: neutral protamine Hagedorn
OAD: oral antidiabetic drug
PP: per protocol
PYE: patient-year of exposure
R: event rate per patient-year of exposure
SD: standard deviation
SDS: standard deviation score
SMBG: self-measured blood glucose
T2D: type 2 diabetes
U: unit of insulin
