# Supplementary material for: A 26-week, randomized trial of insulin detemir versus NPH insulin in children and adolescents with type 2 diabetes (iDEAt2)
Source: Eur J Pediatr. 2018 Jul 16;177(10):1497–503. doi: 10.1007/s00431-018-3205-z (PMC6153598; doi:10.1007/s00431-018-3205-z)
Supplement: Supplementary file 1 — (PDF 195 kb) [file 431_2018_3205_MOESM1_ESM.pdf]

Supplementary Material

A 26-week, randomized trial of insulin detemir versus NPH insulin in children and adolescents with type 2 diabetes (iDEAt2)

Mark D. Wheeler, Margarita Barrientos-Perez, Fu-Sung Lo, Bo Liang, Alison Lunsford, Ólöf Thórisdóttir, Nehama Zuckerman-Levin

Corresponding author: Mark Wheeler, The University of Arizona Department of Pediatrics, 1501 N. Campbell Avenue, Room 3301, PO Box 245073, Tucson, Arizona 85724, USA**;** mwheeler@peds.arizona.edu

Supplementary Table 1. Basal insulin titration algorithm

**Basal insulin dose adjustments**		
**Mean of three pre-breakfast or pre-dinner SMBG values***		
**mmol/L**	**mg/dL**	**Dose adjustment (U)**
4.0–6.0	71–108	No adjustment
6.1–8.0	109–144	+2
8.1–9.0	145–162	+4
9.1–10.0	163–180	+6
>10.0	180	+8
**If one SMBG measurement**		
**mmol/L**	**mg/dL**	**Dose adjustment (U)**
<3.1	<56	−4
3.1–3.9	56–70	−2

*For patients treated on a once-daily regimen, basal insulin titration was done according to the mean of three pre-breakfast SMBG values. For patients treated on a twice-daily regimen, the dose adjustment was based on the mean of three pre-dinner SMBG values. If one SMBG value was missing, the adjustment was performed by the mean of the two SMBG values. If two SMBG values were missing, the adjustment was made on the one SMBG value.

SMBG, self-measured blood glucose; U, unit of insulin.

Supplementary Fig. 1. CONSORT diagram for the clinical trial

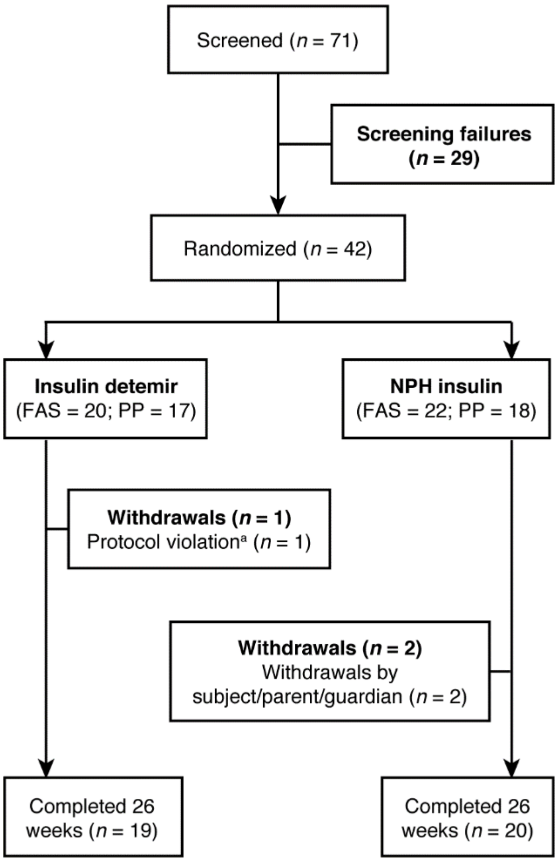

[a]Protocol violation consisted of noncompliance with the protocol resulting in persistent hyperglycemia despite initiation of rescue medication.

FAS, full analysis set: includes all randomized subjects; NPH, neutral protamine Hagedorn; PP, per protocol set: includes all randomized subjects who had been treated for at least 12 weeks and not violating any of the inclusion/exclusion criteria.
